# Barriers and facilitators to STI and HIV testing among African and Caribbean heritage communities in high-income countries mapped to the behaviour change wheel: a scoping review

**DOI:** 10.1186/s12889-026-28379-w

**Published:** 2026-07-10

**Authors:** Temilola Adeniyi, Christie Cabral, Jeremy Horwood

**Affiliations:** 1 National Institute for Health and Care Research, Health Protection Research Unit (HPRU) in Behavioural Science and Evaluation, Population Health Sciences, Bristol Medical School, University of Bristol, Bristol, UK; 2 Population Health Sciences, Bristol Medical School, University of Bristol, Bristol, UK; 3 National Institute for Health and Care Research Applied Research Collaboration West (NIHR ARC West), Bristol, UK; 4 Centre for Academic Primary Care (CAPC), Bristol Medical School, University of Bristol, Bristol, UK

**Keywords:** Sexually Transmitted Infections (STIs), STI Testing Barriers, COM-B Model, Health Disparities, African and Caribbean Communities, Public Health Interventions, Health Equity, High-Income Countries, Behaviour Change, Culturally Relevant Health Services

## Abstract

**Background:**

African and Caribbean communities in high-income countries face disproportionate sexually transmitted infection (STI) risks. In the US, the gonorrhoea rate among non-Hispanic Blacks is 7.7 times greater than that among non-Hispanic Whites, and the chlamydia rate is 5.6 times greater. In the UK, black Caribbeans have the highest gonorrhoea and chlamydia rates among all ethnic minority groups. Identifying barriers to and facilitators of HIV/STI testing is crucial for developing effective interventions. This scoping review maps current evidence on multilevel factors influencing STI testing behaviours among these populations onto the COM-B (Capability, Opportunity, Motivation-Behaviour) model, which posits that capability (i.e., knowledge/skill), opportunity (i.e., social and environmental influence), and motivation (i.e., confidence/beliefs) are essential for engaging in a behaviour (i.e., HIV/STI testing).

**Methods:**

MEDLINE and Embase were searched for studies published between 2013 and 2024 on STI testing barriers and facilitators among African and Caribbean populations in high-income countries. Qualitative, quantitative, and mixed-methods studies were included. The titles/abstracts were screened, the data were charted, and the findings were synthesised via COM-B as an organizing framework.

**Results:**

Fifty-eight studies were included. The key capability barriers were low STI knowledge and language difficulties. Social opportunity barriers included stigma, discrimination, and lack of support. Clinic times and locations impeded physical opportunities. The motivation barriers were fear of positive results, cost, risk perception, confidentiality concerns, and competing priorities. Key facilitators included awareness initiatives, treatment knowledge (capability), supportive networks, outreach (social opportunity), free testing, convenient options (physical opportunity), and risk perceptions, relationships, and incentives (motivation).

**Conclusion:**

This review highlights the complex interplay of COM-B factors influencing STI testing among African and Caribbean heritage communities, drawing attention to pervasive stigma and socioeconomic barriers. Multilevel interventions should enhance capability through education, opportunity via community coproduction and convenient testing, and motivation by addressing stigma and leveraging facilitators. Integrating an intersectionality lens and evaluating community-driven approaches are future directions for promoting sexual health equity.

**Supplementary Information:**

The online version contains supplementary material available at https://doi.org/10.1186/s12889-026-28379-w.

## Background

Sexually transmitted infections (STIs) are a major global public health concern, contributing significantly to the burden of disease worldwide. According to the World Health Organization, more than 1 million STIs are acquired every day, with far-reaching health consequences if left untreated [1]. The impact of STIs is particularly pronounced among certain populations, including African and Caribbean heritage communities in high-income countries [1].

Despite greater resources and access to healthcare in high-income countries, African and Caribbean heritage communities disproportionately bear the burden of STIs such as HIV, chlamydia, and gonorrhoea in high-income countries compared with the general population [2–7]. In the United States, the gonorrhoea rate among African Americans is 7.7 times greater than that among non-Hispanic whites, and the chlamydia rate is 5.6 times greater [8]. Similarly, in the United Kingdom, black Caribbeans have the highest gonorrhoea and chlamydia rates among all ethnic minorities [9].

Late STI diagnosis is common among Africans, enabling ongoing transmission [10]. Undiagnosed STIs further increase the risk of HIV acquisition [11], perpetuating a vicious cycle of infection and transmission within these communities. Some studies provide insights into the barriers faced by African and Caribbean heritage communities in accessing healthcare services in Europe, including language barriers, cultural differences, and a lack of awareness [5, 12]. These disparities underscore the urgent need to address the unique challenges faced by African and Caribbean heritage communities in accessing sexual health services. Also, these disparities are not solely the result of individual-level factors but are underpinned by broader structural determinants, including structural racism, socioeconomic marginalisation, and the enduring impacts of colonisation, which shape healthcare access, institutional trust and stigma thereby influencing STI testing behaviours [13, 14].

Prior systematic reviews have examined healthcare access barriers among migrant populations in Europe, often with a focus on HIV testing and care [15, 16]. However, no reviews have focused specifically on STI testing barriers among African and Caribbean heritage communities living in high-income countries.

To enhance the understanding of the complex factors influencing STI testing, the COM-B (Capability, Opportunity, Motivation-Behaviour) model provides a structured framework to identify key facilitators and barriers. The COM-B model posits that a behaviour (B), e.g., STI testing, is influenced by an individual having the capabilities (C) physically (skills) and psychologically (knowledge); opportunity (O) social (societal influence) and physical (environmental resources); and motivation (M) automatic (emotion) and reflective (beliefs, intentions) [16, 17]. The COM-B model has been effectively utilised in various healthcare settings to understand behaviour change, including in the context of STI testing [15, 16]. By applying the COM-B model [16], this review will identify key capability, opportunity, and motivation barriers and facilitators influencing STI testing uptake in these populations.

This scoping review aims to systematically search, map, and synthesise the existing evidence on barriers to and facilitators of STI testing, including HIV testing experienced by African and Caribbean heritage communities in high-income countries, using the COM-B model of behaviour change to inform the development of targeted interventions to improve STI testing uptake and reduce disparities.

## Methods

### Study design

**Fig. 1:**
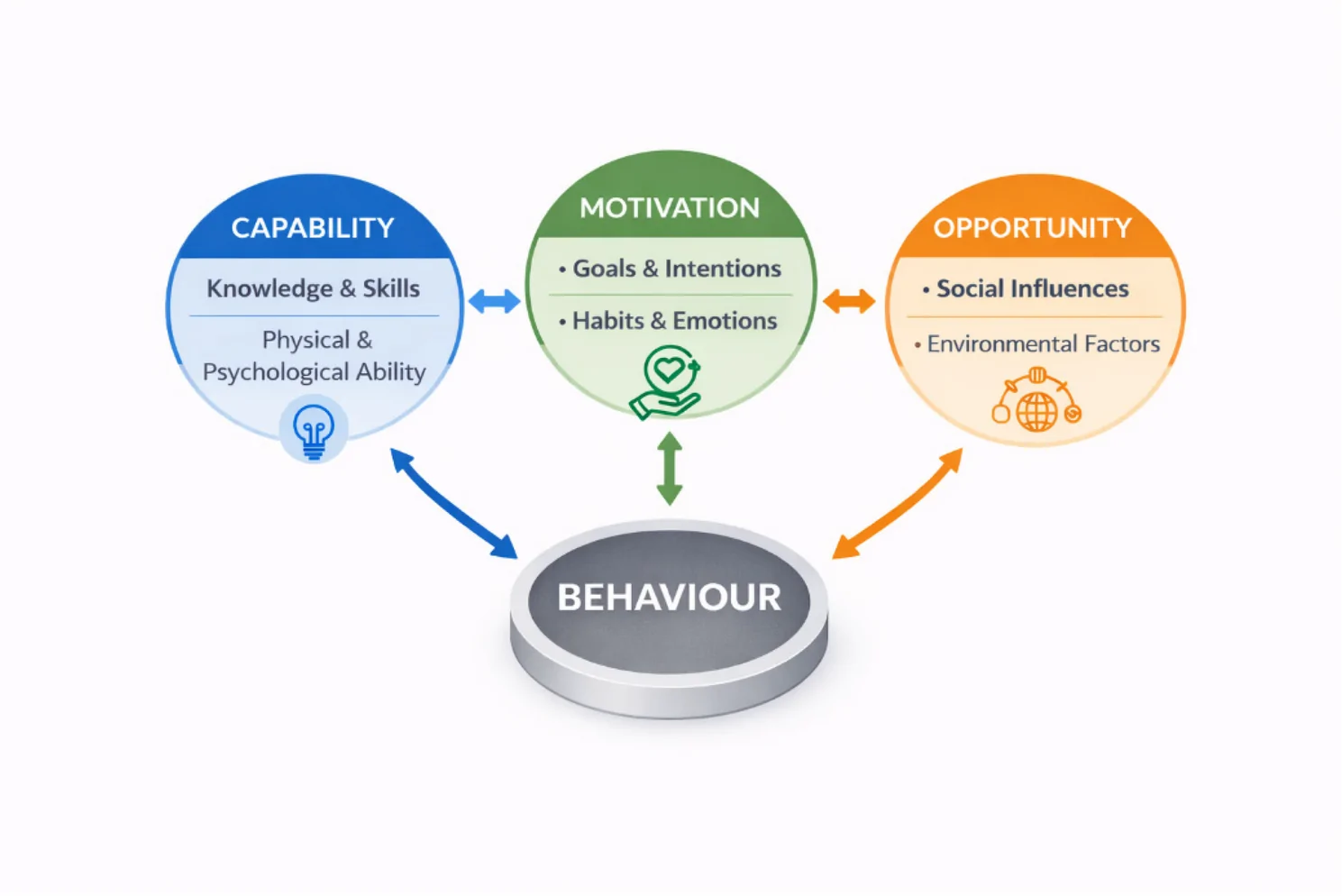
The Capability–Opportunity–Motivation Behaviour (COM-B) model.

This scoping review was conducted following methodological guidance from the Joanna Briggs Institute [18]. A scoping approach was appropriate given the breadth of the research area and the objective to map barriers and facilitators of STI testing onto the COM-B model. Fig. 1.

### Search strategy

To develop a comprehensive search strategy, a research librarian was consulted to identify relevant key terms and medical subject headings (MeSH) related to the study population (Africans and Caribbeans), phenomenon of interest (barriers and facilitators), outcome (STI/HIV testing) and context (high-income countries). The search strategy was limited by year (2013–2024) to reflect contemporary sexual health policy contexts, service delivery models and testing technologies in high-income countries and language (English).

Using the identified terms and criteria, a comprehensive search was conducted in two major databases (Embase and Medline), which were selected based on their broad coverage of relevant literature. Grey literature sources were explored to identify potentially relevant studies, however, no eligible studies meeting the inclusion criteria were identified. The final synthesis therefore focused on peer-reviewed literature to ensure methodological transparency and sufficient reporting detail. The search strategy is presented in the additional file (see additional file 2). Upon completion, the searches from each database were documented and imported into a specific folder in the EndNote X7 software tool for managing bibliographies, citations, and references. Thereafter, all duplicates were removed. The remaining records were exported to Rayyan for title and abstract screening and were shared with the research team.

**Fig. 2:**
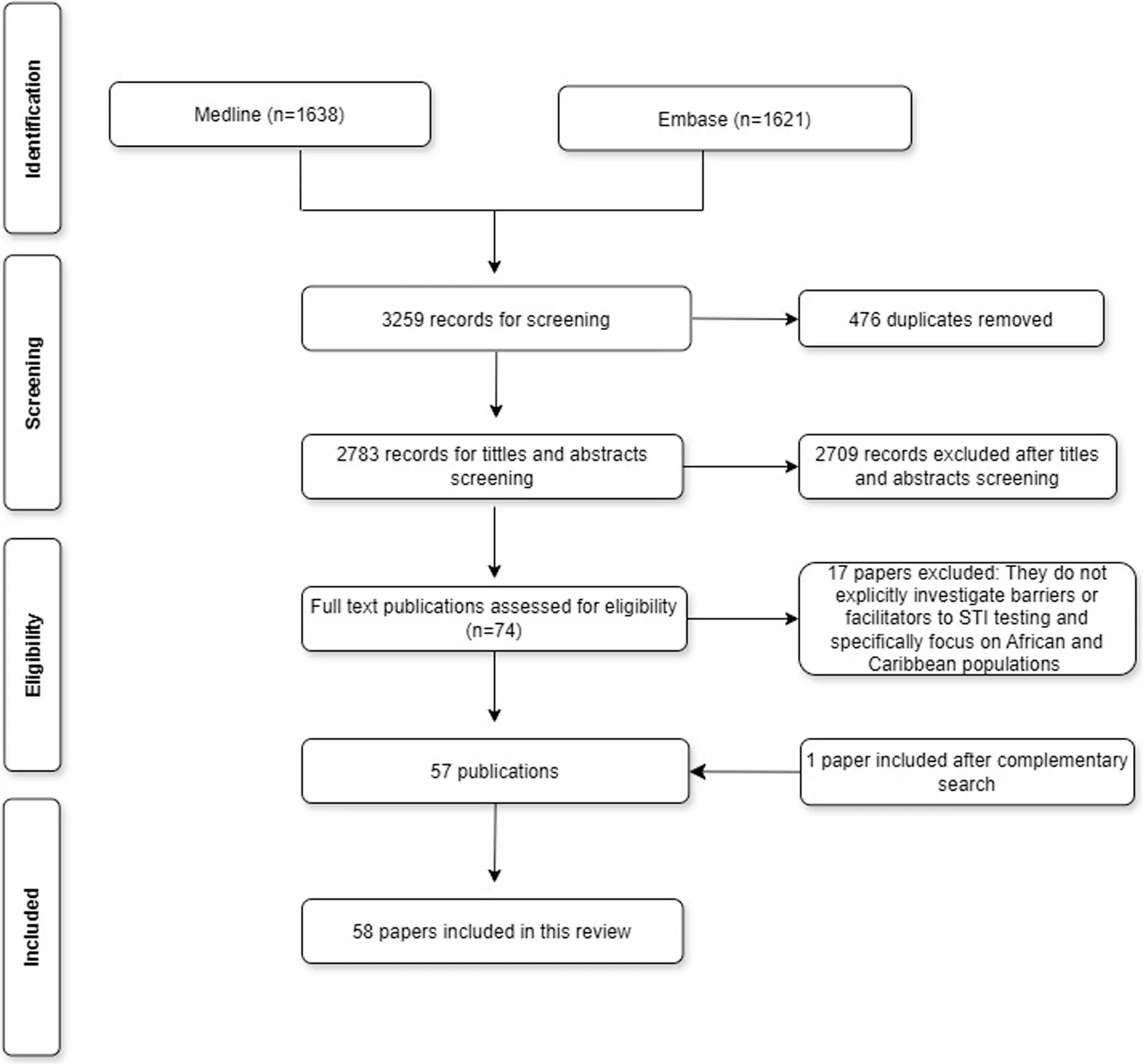
PRISMA flow diagram showing the study selection process

The first author, TA, reviewed all titles and abstracts for inclusion eligibility based on the defined inclusion and exclusion criteria using RAYYAN software. The first 200 records were independently double screened blindly for inclusion by JJ and CC (100 each). TA, JJ, and CC discussed cases where different decisions about inclusion had been made or where there was uncertainty about inclusion. This discussion continued until a consensus was reached about the inclusion criteria. (see Fig. 2)

### Eligibility criteria

Studies were included in this scoping review if they investigated barriers or deterrents and/or facilitators to STI testing, including HIV testing among African and Caribbean heritage communities or black diasporic populations in high-income countries and met the eligibility criteria outlined in the additional file 1.

### Data extraction

A data extraction form in Excel was used to extract relevant data from the included studies via TA, and the data were checked for accuracy by JH and CC. Inconsistencies were resolved through discussion. The extracted information included the following elements: author(s), paper title, year of publication, country, population size, study design, methods, theoretical framework (if applicable), barriers to HIV/STI testing, facilitators of HIV/STI testing, types of stigma (if reported), limitations, and recommendations for future research. See Table 1 for the summary of barriers and facilitators across the COM-B components.

**Table 1:** Summary of barriers and facilitators across the COM-B and TDF components

COM-B Component	TDF	Barrier	Source	Facilitator	Source
Capability	Knowledge	Low STI/HIV knowledge and awareness	[22, 37, 38, 45, 62, 64, 66–68, 77]	STI/HIV education and awareness	[35, 57, 64, 68]
		Language Difficulties	[46, 61, 72, 76]	Understanding STI treatment benefits	[24, 36, 51, 53, 55, 65, 76]
Social Opportunity	Social Influences	STI/HIV Stigma	[20, 21, 25, 26, 28, 29, 36, 39–41, 45–47, 50, 54, 59, 64, 65, 67, 68, 71, 75, 77]	Supportive social networks	[22, 23, 25, 26, 28, 32, 35, 36, 43, 47, 53, 64, 68]
		Lack of Community Support	[22–24, 36, 38, 41, 48, 54, 55, 59, 64, 75]	Community outreach and partnerships	[36, 38, 40, 64, 68]
		Discrimination	[20, 52, 63, 67–69, 76]	Absence of discrimination	[19, 34, 40]
		Cultural norms and expectations	[40, 64, 66, 71, 77]	Situational opportunity	[44, 64]
		Inadequate Provider Training and Support	[64]	Cultural sensitivity training for providers	[48, 64, 75]
		Gender differences	[29, 57, 64]		
Physical Opportunity	Environmental Factors	Poverty/cost issues	[19, 25, 32, 33, 36, 37, 44, 46, 47, 49, 50, 68, 69, 72, 74, 75]	Free/low-cost testing	[42, 47, 53, 75]
		Inadequate testing facilities and services	[19, 27, 29, 47, 50, 53, 67, 75, 76]	Convenient testing locations/options	[24, 32, 33, 38, 41, 52, 64, 75]
		Increased HIV/STI risk	[20, 28, 51, 54, 55]	Confidentiality assurances	[33, 48, 64, 75]
Motivation	Emotions	Fear/anxiety about testing or results	[21, 24, 25, 27, 28, 30, 33, 35, 36, 50, 52–54, 64, 65, 68]	Perceived risk/suspected exposures	[21, 24, 28, 51, 53, 60]
	Beliefs about consequences	Anticipated negative consequences	[46, 49, 69, 74]	New relationship/partners	[24, 28, 34, 55]
		Confidentiality concerns	[19, 30, 32, 50, 53, 64, 72, 74]	Incentives for testing	[36, 40, 41]
		Low-risk perception	[21, 24, 26, 29, 30, 32, 44, 50, 53, 62, 64, 67, 71, 76]	Positive attitudes toward testing	[34, 53, 55, 57]
	Goals and intentions	Competing priorities	[48, 50, 64]		

### Data synthesis

In synthesising the findings, barriers and facilitators were deductively mapped onto the COM-B (Capability, Opportunity, Motivation - Behaviour) framework [17] to understand the mechanisms influencing HIV/STI testing behaviour in these populations.

The initial mapping of barriers and facilitators to COM-B domains was undertaken by TA. Coding decisions and domain allocations were discussed with CC and JJ to ensure conceptual consistency and transparency. Where uncertainties arose, consensus was reached through discussion and refinement of domain definitions.

**Fig. 3:**
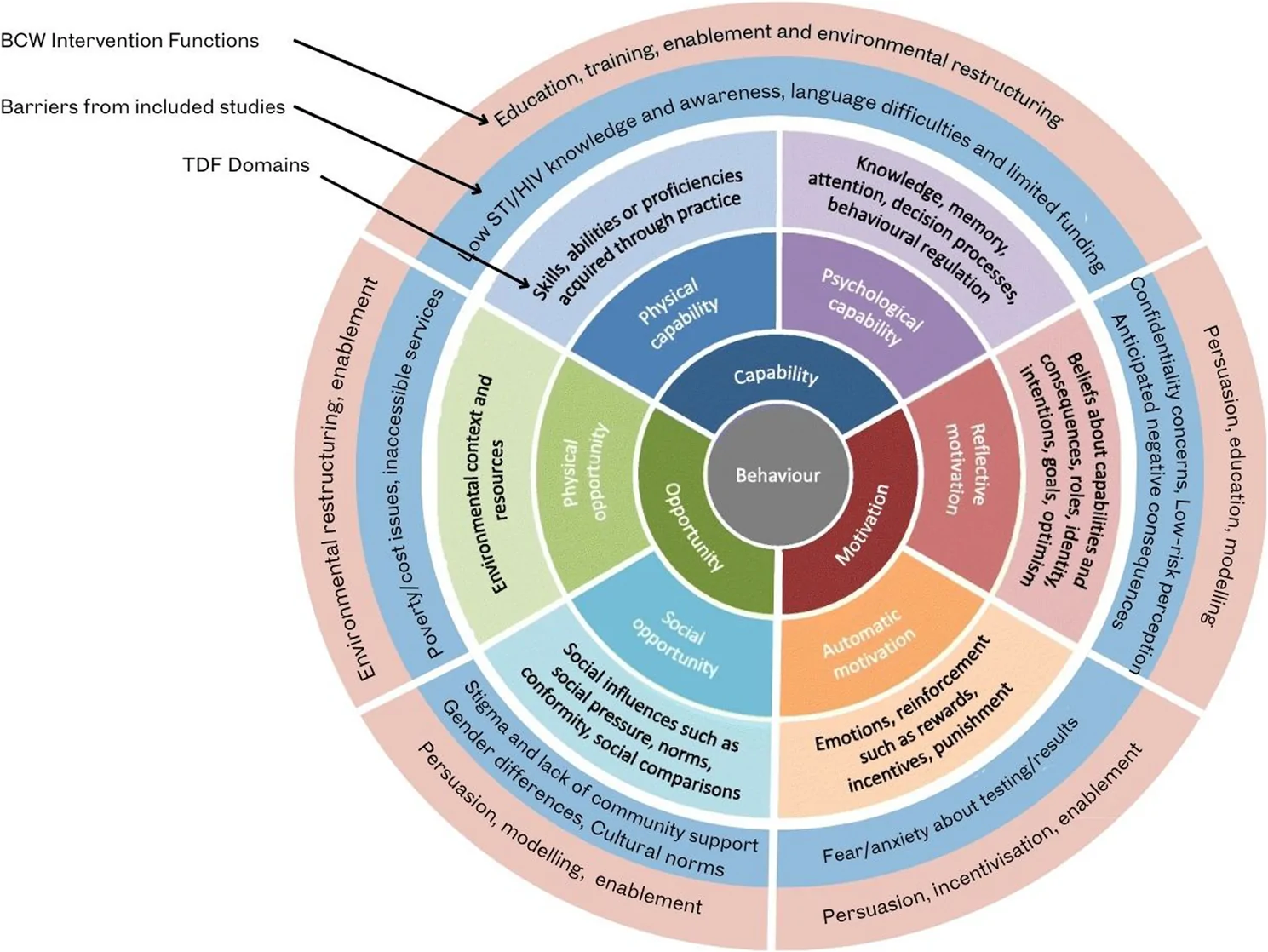
Barriers to HIV/STI testing among African and Caribbean heritage communities mapped onto COM-B, TDF, and BCW intervention functions. Adapted from Michie et al. (2011), with Theoretical Domains Framework domains adapted from Cane et al. (2012)

**Fig. 4:**
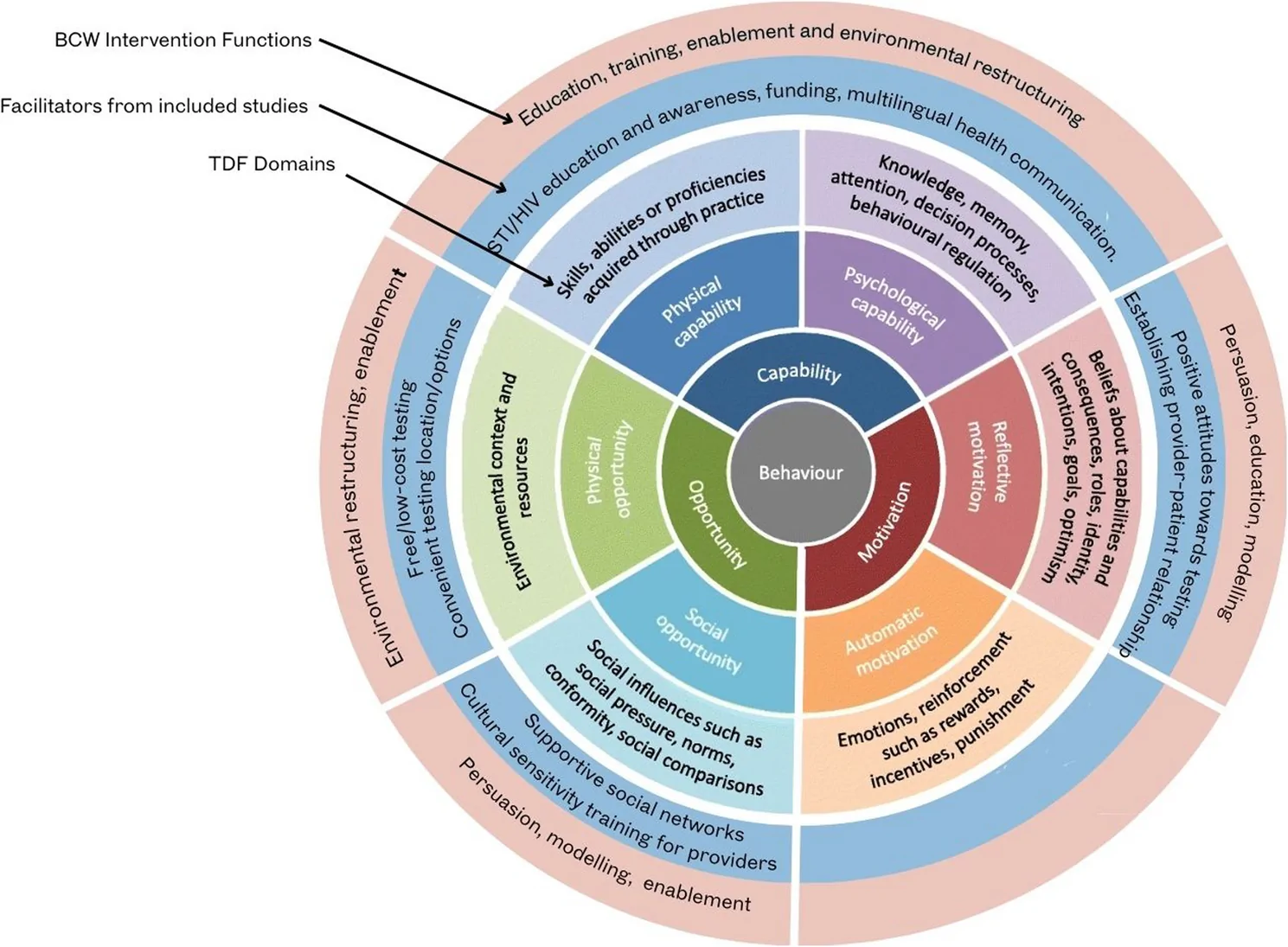
Facilitators for HIV/STI testing among African and Caribbean heritage communities mapped onto COM-B, TDF, and BCW intervention functions. Adapted from Michie et al. (2011), with Theoretical Domains Framework domains adapted from Cane et al. (2012)

Given the range of testing-related behaviours represented in the included studies, determinants were synthesised at the overarching behavioural level of engagement in STI testing. While some studies referenced theoretical frameworks, theoretical application was inconsistent and varied in depth, limiting meaningful theoretical comparison. We adopted a staged, theory-informed analytic approach consistent with the Behaviour Change Wheel (BCW) framework. First, we used the COM-B model to provide an overarching behavioural diagnosis, categorising the barriers and facilitators to testing in terms of Capability, Opportunity, and Motivation, consistent with COM-B’s role as the core behavioural system within the BCW. We then applied the Theoretical Domains Framework (TDF) to refine these COM-B categories, allowing us to map the determinants onto its more detailed behavioural domains where the available data supported this level of granularity. As the final step, the BCW was used to identify the intervention functions relevant to the mapped COM-B/TDF determinants, highlighting the types of behaviour change strategies for addressing the barriers and strengthening the facilitators identified in the review. Figures 3 and 4 present the COM-B, TDF, and BCW intervention functions for transparency and to illustrate conceptual alignment with the BCW approach.

## Results

### Study characteristics

The fifty-eight included studies represented research conducted predominantly in the United States (*n* = 40) [19–58], the United Kingdom (*n* = 8) [59–66], and Canada (*n* = 4) [67–70], with some studies also from Australia (*n* = 2) [71, 72], Germany (*n* = 1) [73], France (*n* = 1) [74], Ireland (*n* = 1) [75] and China (*n* = 1) [76]. The studies focused on African and/or Caribbean populations, communities, and immigrants residing in these high-income countries.

In terms of study design, there were thirty-one qualitative studies utilising methods such as focus groups and interviews, twenty-three quantitative studies including surveys and analyses of national datasets, and four mixed-methods studies combining qualitative and quantitative approaches. Most studies were descriptive (qualitative or cross-sectional), examining barriers and facilitators to HIV/STI testing, while four studies evaluated or piloted interventions, including community-based initiatives [48, 50, 58] and digital intervention [70]. The qualitative studies allowed for in-depth exploration of barriers, facilitators, and experiences related to HIV/STI testing from the perspectives of the populations of interest. The sample sizes ranged from 14 to 57 participants. The quantitative studies had larger sample sizes ranging from 14 to 10,397 participants. A few studies have applied theoretical frameworks such as the PEN-3 model [30], comprehensive health-seeking and coping paradigm [27], Andersen’s model of health care utilisation [32, 47], intersectionality theory [67, 69], responsive evaluation framework [50], socioecological model [49, 53, 56, 68], COM-B model [59], and ecodevelopmental theory [22].

Several studies have targeted key subpopulations, such as men who have sex with men [20, 34, 42, 47, 52–54, 63], youth/young adults [24, 36, 40, 42, 47, 50, 51, 53, 56–58, 66, 77], HIV-serodiscordant couples [19], and individuals with higher HIV/STI risk profiles [49, 51, 55]. Fifty-three studies focused on HIV testing specifically [20–39, 41–49, 51–57, 59–63, 65, 67–76], one on blood-borne viruses (HIV, Hepatitis B and C) [64] and four examined broader STI testing [40, 50, 58, 66]. Eleven studies examined past HIV testing behaviour or uptake [25, 34, 39, 43, 48, 53, 55, 57, 68, 75, 76], while others explored intentions, preferences or experiences related to HIV/STI testing. A table outlining the key characteristics of the included studies is presented in the supplementary file. (See additional file 3).

### Barriers and facilitators to HIV/STI testing

#### Capability barriers

i. Low HIV/STI knowledge and awareness: Across multiple studies, a lack of knowledge and awareness about HIV/STI transmission, testing procedures, and available services was identified as a key barrier for African and Caribbean heritage communities [22, 37, 38, 45, 62, 64, 66–68, 77]. Limited health literacy, campaigns, and advocacy have contributed to misconceptions about STI risk and susceptibility [30, 31, 48, 64, 71]. The specific knowledge gaps highlighted included being unaware of testing locations [61, 75] and unfamiliar with self-testing methods [27, 34, 38, 54, 59, 61, 64–66].
ii. Language Difficulties: Language difficulty was identified as a barrier to accessing information about testing, understanding test results, and communicating with healthcare providers [46, 61, 72, 76].

#### Capability facilitators

i. HIV/STI education and awareness: Increasing knowledge through community-based HIV/STI education and awareness initiatives and increased funding facilitated testing across studies [35, 57, 64, 68]. The incorporation of culturally relevant messaging and videos has helped enhance the resonance and impact of these awareness efforts [38, 41].
ii. Understanding HIV/STI treatment benefits: For those aware of current HIV/STI treatment effectiveness, studies have shown that this knowledge motivated testing by reducing the perceived consequences of a positive diagnosis [24, 36, 51, 53, 55, 65, 76]. Evidence suggests that testing uptake can be enhanced through understanding HIV/STI transmission mechanisms, coupled with clear instructions for self-testing kits [27, 57, 65, 76].

#### Opportunity barriers

-  ❖ Social opportunity barriers

i. HIV/STI stigma: stigma surrounding HIV/STIs emerged as a pervasive social opportunity barrier across numerous studies [20, 21, 25, 26, 28, 29, 36, 39–41, 45–47, 50, 54, 59, 64, 65, 67, 68, 71, 75, 77]. Different forms of stigma, including perceived stigma and felt stigma from communities [30, 39, 66, 67, 76]; experienced stigma and discrimination from healthcare providers [48, 53, 55, 64, 68, 72, 75]; intersectional stigma, where the impact of HIV/STI stigma intersects with other forms of stigma, including racism, xenophobia, homophobia, and stigma towards sex work; migration status; drug and substance use; and gender identities [20, 28, 29, 54, 55]; and institutionalised stigma within healthcare systems [20, 67, 75], have been highlighted. Stigma led to fears of judgment, rejection, and fear of disclosing status.
ii. discrimination: in addition to HIV/STI stigma, studies have identified experiences of racial/ethnic discrimination [20, 52, 63, 67–69, 76], homophobia [20, 52], cultural insensitivity [64], and discrimination related to immigration status [46, 49, 69] as key social barriers restricting opportunities for testing access. Mistrust in healthcare systems due to historical injustices towards minority groups also manifested as a barrier [55, 66, 67, 77].
iii. Lack of Community Support: Multiple studies highlighted how isolation, shame, and lack of supportive social networks undermine testing [22–24, 36, 38, 41, 48, 54, 55, 59, 64, 75]. This lack of community acceptance and affirmation hampered opportunities for testing. Poor links with community organisations were identified as deterrents [64]. Negative stories such as HIV as a ‘death sentence’ told by community members deterred people from testing [22, 48, 72].
iv. Cultural Norms and Expectations: Cultural norms and expectations influenced testing behaviours, with some norms serving as barriers (e.g., prohibitions around discussing sexual health and assumptions of immoral behaviour) [40, 64, 66, 71, 77]. Religious and moral beliefs also factored into shaping norms around testing in certain communities, associating HIV testing with perceived immoral behaviour [30, 64, 66, 68, 77].
v. Gender differences: Gender differences emerged regarding barriers, with studies highlighting how African/Caribbean women faced additional barriers such as gender power dynamics, enacted stigma, and cultural expectations around femininity norms such as expectations of sexual purity [29, 57, 64]. Lower engagement with testing among some men was linked to gendered norms that framed healthcare-seeking as a sign of weakness [64].
vi. Inadequate Provider Training and Support: Health providers had limited training on proper STI guidelines and counselling [64]. This lack of comprehensive education and support for healthcare professionals led to missed opportunities for STI testing, inadequate pre- and post-testing counselling, and suboptimal patient experiences when accessing testing services.

-  ❖ Physical opportunity barriers

i. Poverty/cost issues: Financial barriers due to a lack of health insurance coverage, poverty, and costs associated with testing services impeded physical opportunities for testing [19, 25, 32, 33, 36, 37, 44, 46, 47, 49, 50, 68, 69, 72, 74, 75].
ii. Inadequate testing facilities and services: Geographic inaccessibility of testing facilities, lack of information on testing locations, and lack of transportation options restricted testing opportunities, especially in rural and low-income areas [19, 27, 29, 47, 50, 53, 67, 75, 76]. Other access barriers include inconvenient clinic hours, excessive wait times, fragmentation of services, and lack of walk-in appointments [19, 32, 50, 63, 64, 75]. Concerns about a lack of privacy in testing locations and being seen in public appeared to be barriers ([19, 24, 38, 41, 47, 53, 59, 64]. This prevented some from accessing testing services through regular healthcare channels.
iii. High-risk subgroups: For subgroups such as men who have sex with men and serodiscordant couples, drug and substance users had limited access to STI testing services in their physical environments, especially when they were homeless or lived in areas with high drug use prevalence [20, 28, 51, 54, 55].

#### Opportunity facilitators

- ❖ Social opportunity facilitators

i. Supportive social networks: Having supportive friends, family members, being married or in a cohabiting relationship, peers and community ties that encouraged testing, emerged as important social facilitators [22, 23, 25, 26, 28, 32, 35, 36, 43, 47, 53, 64, 68]. Involving trusted peers and community leaders in testing promotion enhanced opportunities [35, 43].
ii. Community outreach and partnerships: Successful interventions involved community organisations, faith-based groups, businesses, and culturally relevant venues to integrate testing and outreach [36, 38, 40, 64, 68].
iii. Absence of discrimination: Experiencing no recent discrimination created a supportive social environment that enhanced the opportunity for individuals to access STI testing services without fear or apprehension [19, 34, 40]. When individuals feel safe and respected, they were more likely to seek out and utilise available healthcare services, including STI testing [19, 40, 64].
iv. Situational Opportunity: Prompting that individuals seek STI testing as part of immigration requirements increased testing [44]. Similarly, being offered testing by a healthcare provider during a medical visit provided an opportunity for individuals to access testing services conveniently [44, 64].
v. Cultural sensitivity training for providers: This enhanced the ability of healthcare providers to understand and respond effectively to the cultural needs and preferences of African and Caribbean heritage communities [48, 64, 75]. This training equipped providers with the knowledge, skills, and confidence to deliver STI testing services in a culturally competent manner.

- ❖ Physical opportunity facilitators

i. Free/low-cost testing: Unsurprisingly, free or low-cost HIV/STI testing services facilitated uptake by minimizing financial barriers across studies [42, 47, 53, 75]. This included free community-based testing events and the distribution of self-test kits without cost to users.
ii. Convenient testing locations/options: Bringing testing to convenient community locations such as businesses, clinics, events, and facilities improved physical access opportunities [24, 32, 33, 38, 41, 52, 64, 75]. Short waits for results encouraged testing [27, 37]. Adopting innovative approaches such as self-testing, routine opt-out testing, and self-sampling kits further enhanced convenience [42, 46, 48, 55, 59, 65, 70, 72].
iii. Confidentiality assurances: Clear assurances of confidentiality and privacy protections increased comfort and facilitated testing [33, 48, 64, 75]. Providing separate, private testing spaces within healthcare and community settings also helped address confidentiality concerns as a facilitator [48].

#### Motivation barriers

- ❖ Automatic motivation barriers

i. Fear/anxiety about testing or results: Multiple studies have indicated that fear represents a key motivation barrier, including fear of positive test results, fear of needles/blood draws, and generalized anxiety around being tested [21, 24, 25, 27, 28, 30, 33, 35, 36, 50, 52–54, 64, 65, 68]. Anticipated stigma and worries about health/life implications contributed to these fears.

- ❖ Reflective motivation barriers

i. Anticipated negative consequences: Participants frequently cited concerns about negative repercussions and life disruptions because of testing positive, which undermined their motivation to be tested [46, 49, 69, 74]. The anticipated consequences included HIV stigma, relationship conflicts/dissolution, impacts on immigration status, and the loss of employment/housing opportunities. Some participants indicated that they had prior negative experiences with testing (e.g., specific counsellors/healthcare providers) [33, 34].
ii. Low-Risk Perception: Even when aware of STIs, many individuals within these communities do not perceive themselves at risk, which reduced their motivation to be tested [21, 24, 26, 29, 30, 32, 44, 50, 53, 62, 64, 67, 71, 76]. Low-risk perceptions were linked to assumptions such as considering oneself low risk due to monogamous relationships [25, 30, 31], a lack of visible symptoms [54], or young age [25].
iii. Confidentiality concerns: In addition to privacy opportunity barriers, confidentiality emerged as a distinct motivation barrier where distrust and doubts about ensuring confidentiality discouraged testing [19, 30, 32, 50, 53, 64, 72, 74]. This stemmed from fears of test results becoming public, distrust in the ability of the healthcare system to protect their personal information, and associations with HIV/STIs in smaller communities.
iv. Competing priorities: For some individuals, low prioritisation of sexual health relative to basic needs such as housing/employment and immediate life stressors reduced their motivation to seek testing [48, 50, 64]. Drug and substance users had competing priorities or a lack of self-efficacy in managing their health [55]. This motivation barrier frequently intersects with the socioeconomic disadvantages faced by the population.

#### Motivation facilitators

- ❖ Reflective motivation facilitators

i. Perceived risk/suspected exposures: Across studies, individuals’ motivation for testing increased when they perceived themselves to be at higher HIV/STI risk through experiences such as recent unprotected sexual encounters, symptoms of infection, previous STI diagnosis, pregnancy, having multiple partners, or suspected exposure events [21, 24, 28, 51, 53, 60].
ii. New relationships/partners: The formation of new sexual partnerships has emerged as a common motivator and catalyst for seeking testing [24, 28, 34, 55]. This enabled partners to establish mutual knowledge of STI status before the initiation of sexual activity.
iii. Incentives for testing: Several studies have shown that providing small monetary incentives or gift cards facilitated motivation and made people follow-through with testing [36, 40, 41].
iv. Positive attitudes toward testing: Some individuals were motivated to seek STI testing because of their positive attitudes and beliefs about the importance of regular testing for maintaining good health [34, 53, 55, 57]. These intrinsic factors, such as the belief that testing is a responsible and necessary part of self-care, facilitated regular testing behaviour. Older adults demonstrated a greater level of awareness of sexual health issues and understood the need to test themselves [25, 57].
v. Establishing Provider-Patient Relationship: Some studies reported that respectful, non-judgemental interactions with healthcare providers increased confidence in confidentiality and reduced concerns about stigma, thereby encouraging engagement with HIV/STI testing services [24, 33, 75].

Building on the COM-B behavioural diagnosis, we extended the analysis using the Behaviour Change Wheel (BCW) to identify intervention functions and relevant policy categories aligned with the barriers and facilitators identified across capability, opportunity and motivation. Capability-related barriers, such as low HIV/STI knowledge and language difficulties, mapped primarily onto the intervention functions of education, training and enablement, while capability facilitators such as improved awareness and understanding of treatment aligned with education and persuasion.

Opportunity-related barriers, including stigma, discrimination, inadequate provider training, cost and limited service accessibility, corresponded to environmental restructuring, modelling and enablement. Opportunity facilitators such as supportive social networks, culturally sensitive providers, community outreach, free testing and convenient testing options aligned with modelling, enablement and environmental restructuring. Motivation-related barriers, including fear, anxiety, anticipated negative consequences, confidentiality concerns, low perceived risk and competing priorities, mapped onto persuasion, incentivisation and modelling, while facilitators such as perceived risk, new relationships, incentives and positive attitudes towards testing aligned with persuasion, incentivisation and education.

At the policy level, the findings suggest relevance for communication and marketing, guidelines and workforce development, service provision and environmental/social planning, and fiscal measures to address structural barriers and support improved access to testing.

## Discussion

This scoping review synthesised evidence on the barriers to and facilitators of STI testing among African and Caribbean heritage communities in high-income countries. By applying the COM-B model, we identified factors influencing testing behaviours across capability, opportunity, and motivation dimensions. Capability barriers were dominated by low HIV/STI knowledge and language difficulties, whereas social opportunity barriers included stigma, discrimination, and inadequate social support systems. Physical opportunity barriers centred on inaccessible testing services, while motivation barriers were often related to fear of positive results, confidentiality concerns, and low perceived risk of infection. Facilitators included community-driven HIV/STI education programmes, supportive social networks, culturally relevant outreach initiatives, and free or low-cost testing services. These findings highlight the multifaceted challenges these communities face, necessitating interventions to improve testing uptake.

This review also revealed several cross-cutting themes and intersecting influences. Notably, intersectional influences such as race, ethnicity, gender, and socioeconomic status intensified the barriers to HIV/STI testing, particularly for vulnerable subgroups such as women, men who have sex with men, and recent immigrants. Intersectionality theory provides a useful framework for understanding how multiple social identities and systems of oppression interact to shape health inequities [78]. Our findings demonstrate the importance of applying an intersectional approach to HIV/STI testing research and interventions in African and Caribbean heritage communities. For example, recent immigrants described fears related to immigration status or confidentiality, while gendered norms shaped perceptions of risk and testing acceptability. Recognising the intersections of race, ethnicity, gender, sexual orientation, and other social determinants is crucial for developing tailored strategies that address the unique needs and experiences of diverse subgroups.

Cultural norms and expectations played a significant role in shaping testing behaviours among African and Caribbean communities. These findings align with previous research highlighting the impact of cultural taboos and silence around sexual health issues on HIV/STI testing in African communities [79]. Findings on gender differences resonate with the broader literature on gender inequities in sexual health, which highlights how patriarchal norms, intimate partner violence, and limited sexual autonomy disproportionately affect women’s access to HIV/STI services [80–82]. Lower engagement with testing among some African and Caribbean men [64] mirrors global trends of men’s underutilisation of health services influenced by gender norms, stigma and structural barriers [83]. Addressing gender-related barriers requires interventions that challenge harmful masculinity norms, promote women’s empowerment, and engage men in sexual health initiatives.

Limitations of this review include the inclusion of only English-language studies, which may have excluded relevant research from non-English-speaking countries. Variability in study designs, populations, and contexts also presented challenges for direct comparisons and synthesis. Although formal quality appraisal was not conducted, consistent with scoping review methodology, the included studies varied in methodological rigour and reporting depth. In addition, inconsistent specification of testing behaviours and limited use of behavioural theory were identified as gaps in the literature.

Extending the COM-B analysis using the Behaviour Change Wheel highlights several potential intervention pathways for improving HIV/STI testing uptake in African and Caribbean heritage communities. Interventions informed by the review findings could enhance capability through culturally relevant education and clear information about testing and treatment; strengthen opportunity through environmental restructuring, such as community-based testing models, culturally competent providers, convenient and low-cost testing options, and reduced structural barriers; and address motivational barriers through persuasion, modelling and incentives that normalise testing and reduce fear, stigma and confidentiality concerns. At a policy level, the findings also indicate the importance of communication strategies, workforce development, service provision reforms and fiscal measures that improve the accessibility and cultural responsiveness of testing services. These insights provide the foundation for our subsequent co-production work with community members and stakeholders to develop a culturally tailored intervention to increase STI testing among African and Caribbean heritage communities in the UK.

All promotional materials should be co-produced with communities to ensure that they are culturally acceptable, accessible, and available in multiple languages for key audiences. While few included studies explicitly employed community-based participatory research approaches [48, 50], the limited number of community-engaged interventions and the identified barriers related to trust and cultural relevance suggest that greater application of CBPR represents an important gap in the literature. Community-based participatory research (CBPR) [84], which actively engages African and Caribbean heritage communities in all phases of the research process, is essential for ensuring the cultural relevance and sustainability of interventions.

Future research should also prioritise intersectional analyses that examine how various social identities and systems of oppression shape HIV/STI testing experiences and outcomes in these communities. This could involve partnerships with trusted community organisations to co-produce services that meet specific cultural needs, e.g., community-based outreach and testing in convenient and accessible locations. This can also provide social role models with which to overcome social opportunity barriers and increase motivation to test.

Studies should also focus on underserved subgroups within the African and Caribbean heritage communities, such as sex workers and recent migrants, to develop targeted testing strategies that address their unique barriers (e.g., language, legal precarity, disrupted healthcare access, high HIV/STI risk) and leverage relevant facilitators. Community-based outreach and partnerships with trusted organisations serving these subgroups can enhance engagement and participation in research and interventions. Longitudinal research is needed to assess the long-term impacts of interventions on HIV/STI testing uptake, sexual health outcomes, and health equity. Such studies can provide insights into the sustainability and scalability of interventions and identify potential adaptations or modifications needed to maintain effectiveness in diverse contexts.

## Conclusion

In conclusion, this scoping review provides a comprehensive synthesis of the barriers to and facilitators of STI testing among African and Caribbean heritage communities in high-income countries. The application of the COM-B model offers a structured framework for understanding the complex determinants of testing behaviours. The behavioural diagnosis and Behaviour Change Wheel mapping provide a foundation for the development of theoretically informed, community-led interventions to improve STI testing uptake. Importantly, these determinants operate within intersecting social and structural contexts, including racism, migration status, gender norms and socioeconomic disadvantage, highlighting the value of an intersectional lens in intervention design. Policymakers, healthcare providers, and public health practitioners should prioritize the sexual health needs of African and Caribbean heritage communities and work in partnership with communities to develop and implement equitable STI testing strategies.

## Supplementary Information

Supplementary Material 1

Supplementary Material 2

Supplementary Material 3

## Data Availability

All data generated or analysed during this study are included in this published article and its supplementary information files.

## Abbreviations

STIs: Sexually Transmitted Infections
COM: B-Capability, Opportunity, Motivation-Behaviour
MeSH: Medical Subject Headings
CBPR: Community-based participatory research
TDF: Theoretical Domains Framework
BCW: Behaviour Change Wheel
