# Supplementary material for: Barriers and facilitators to STI and HIV testing among African and Caribbean heritage communities in high-income countries mapped to the behaviour change wheel: a scoping review
Source: BMC Public Health. 2026 Jul 10;26:2628. doi: 10.1186/s12889-026-28379-w (PMC13589379; doi:10.1186/s12889-026-28379-w)
Supplement: Supplementary file 1 [file 12889_2026_28379_MOESM3_ESM.docx]

# ▲	Searches	Results	Type	Actions		Annotations
1	exp African Continental Ancestry Group/	97645	Advanced	Display Results	More	
2	(african* or africaan* or caribbean* or afro-caribbean* or afrocaribbean*).ti.	69477	Advanced	Display Results	More	
3	((african* or africaan* or caribbean* or afro-caribbean* or afrocaribbean*) adj (diaspora or heritage or origin or decent or migrant? or immigrant? or communit* or neighbourhood* or neighborhood*)).ab,kf.	4143	Advanced	Display Results	More	
4	exp Blacks/ or Black Person/	97645	Advanced	Display Results	More	
5	(black or blacks).ti. or (black adj3 (men or women or adult? or people? or population or adolesc* or teen* or young* or youth? or old* or elder*)).tw,kf.	69436	Advanced	Display Results	More	
6	Black British*.mp.	125	Advanced	Display Results	More	
7	Black-White.tw,kf.	3137	Advanced	Display Results	More	
8	(black? adj3 asian? adj3 (minorit* or ethnic*)).mp.	800	Advanced	Display Results	More	
9	(landed adj5 (person* or resident* or status)).tw,kf.	9	Advanced	Display Results	More	
10	exp Refugees/	13360	Advanced	Display Results	More	
11	(refugee? or asylum).tw.	17165	Advanced	Display Results	More	
12	exp MINORITY GROUPS/	18158	Advanced	Display Results	More	
13	MINORITY HEALTH/	898	Advanced	Display Results	More	
14	minorit*.tw,kf.	94080	Advanced	Display Results	More	
15	or/1-14	301833	Advanced	Display Results	More	
16	(sexual and reproductive health).mp.	9484	Advanced	Display Results	More	
17	Sexual Health/	2529	Advanced	Display Results	More	
18	exp sexually transmitted diseases/ or sexually transmitted diseases, bacterial/ or chancroid/ or chlamydia infections/ or lymphogranuloma venereum/ or gonorrhea/ or granuloma inguinale/ or syphilis/ or sexually transmitted diseases, viral/ or condylomata acuminata/ or herpes genitalis/ or exp hiv infections/ or papillomavirus infections/	421883	Advanced	Display Results	More	
19	(sex* transmit* adj3 (disease* or infect*)).tw,kf.	37036	Advanced	Display Results	More	
20	(chlamydia or gonorhea or gonorrhoeae or syphilis or (herpes and (genital* or sex*)) or papillomavir* or papilloma vir* or HPV).tw,kf.	138971	Advanced	Display Results	More	
21	HIV infections/	226440	Advanced	Display Results	More	
22	(HIV or human immunodeficien* vir* or human immunedeficien* vir* or human immun* deficien* vir* or acquired immunodeficien* syndrom* or acquired immunedeficien* syndrome* or immun* deficien* syndrom*).tw,kf.	389058	Advanced	Display Results	More	
23	(STI or STIs or STDs).ab.	18544	Advanced	Display Results	More	
24	or/16-23	624577	Advanced	Display Results	More	
25	Mass Screening/	116583	Advanced	Display Results	More	
26	24 and 25	14284	Advanced	Display Results	More	
27	((test* or screen*) adj5 (STI or STIs or STDs or sex* transmit* or venereal disease* or chlamydia or gonorhea or gonorrhoeae or syphilis or (herpes and (genital* or sex*)) or papillomavir* or papilloma vir* or HPV)).tw,kf.	27078	Advanced	Display Results	More	
28	HIV Testing/	949	Advanced	Display Results	More	
29	((test* or screen*) adj5 (HIV or human immunodeficien* vir* or human immunedeficien* vir* or human immun* deficien* vir* or acquired immunodeficien* syndrom* or acquired immunedeficien* syndrome* or immun* deficien* syndrom*)).tw,kf.	41729	Advanced	Display Results	More	
30	or/26-29	69845	Advanced	Display Results	More	
31	(attitude? or barrier? or confidentiality or cultural factor? or deterrent? or distrust* or fear or health disparit* or accessing or "access to" or hesitan* or stigma or mistrust* or misinform* or obstacle? or perceived risk? or perception* or skepticism or social factor? or trust* or willingness).mp.	1983596	Advanced	Display Results	More	
32	(awareness* or choice? or facilitat* or implement* or incentive* or influenc* or motivat* or preference?).mp.	3911635	Advanced	Display Results	More	
33	(takeup or take-up or uptake or up-take or adherence).mp.	657979	Advanced	Display Results	More	
34	or/31-33	5791549	Advanced	Display Results	More	
35	15 and 30 and 34	2194	Advanced	Display Results	More	
