# Supplementary material for: Barriers and facilitators to STI and HIV testing among African and Caribbean heritage communities in high-income countries mapped to the behaviour change wheel: a scoping review
Source: BMC Public Health. 2026 Jul 10;26:2628. doi: 10.1186/s12889-026-28379-w (PMC13589379; doi:10.1186/s12889-026-28379-w)
Supplement: Supplementary file 1 [file 12889_2026_28379_MOESM2_ESM.docx]

|  | **Inclusion Criteria:** | **Exclusion Criteria** |
| --- | --- | --- |
| **Population:** | Studies involving African and Caribbean community, ethnic minorities, or diasporic populations in high-income countries.  * Studies were included if they explicitly reported data on participants identified as African, Caribbean, Black African, Black Caribbean, or African diaspora populations residing in high-income countries.  * Studies of general populations were included only if subgroup data for African or Caribbean participants were reported separately. | Studies not conducted in high-income countries.  Studies involving general populations not specific to people of African and Caribbean heritage* |
| **Phenomenon of Interest:** | Studies were included if they examined barriers, facilitators, determinants or experiences related to uptake of STI testing (including HIV testing where explicitly examined). | Studies focusing solely on treatment outcomes or unrelated health behaviours. |
| **Study Design:** | Quantitative studies (e.g. cross-sectional surveys, cohort studies, trials)  Qualitative studies (e.g. interviews, focus groups)  Mixed methods studies  Pilot studies | Non-empirical publications (editorials, commentaries, protocols, etc.)  Review articles (systematic, scoping or narrative reviews) |
| **Other:** |  | Abstracts for which full text cannot be retrieved  Duplicate publications reporting on same data |

Keywords:

Population terms:

African, Africa, Caribbean, Black, Minority, Migrant, Immigrant, Refugee, Diaspora

Phenomenon terms:

Barrier, Obstacle, Challenge*, Deterrent*, Hindrance, Impediment

Outcome terms:

STI testing, Sexually transmitted infection, HIV testing, HIV screen, Chlamydia test, Gonorrhoea test, Syphilis test, STI service

Context: High-income countries are those that have a gross national income per capita of $13,206 in 2023. Globally, 81 countries qualify for this category.
